# A Cluster Analysis of Constant Ambient Air Monitoring Data from the Kanto Region of Japan

**DOI:** 10.3390/ijerph110706844

**Published:** 2014-07-03

**Authors:** Atsushi Iizuka, Shintaro Shirato, Atsushi Mizukoshi, Miyuki Noguchi, Akihiro Yamasaki, Yukio Yanagisawa

**Affiliations:** 1 Research Center for Sustainable Science and Engineering, Institute of Multidisciplinary Research for Advanced Materials, Tohoku University, 2-1-1 Katahira, Aoba-ku, Sendai, Miyagi 980-8577, Japan; 2 Department of Environmental Systems, Institute of Frontier Sciences, The University of Tokyo, 5-1-5 Kashiwanoha, Kashiwa, Chiba 277-8563, Japan; E-Mails: shintaro_shirato@yy.k.u-tokyo.ac.jp (S.S.); mizukoshi@med.kindai.ac.jp (A.M.); yukio@k.u-tokyo.ac.jp (Y.Y.); 3 Tokyo Metropolitan Industrial Technology Research Institute, 2-4-10 Aomi, Koto-ku, Tokyo 135-0064, Japan; 4 Department of Materials and Life Science, Faculty of Science and Technology, Seikei University, 3-3-1 Kichijoji-kitamachi, Musashino, Tokyo 180-8633, Japan; E-Mails: miyuki_noguchi@yy.k.u-tokyo.ac.jp (M.N.); akihiro@st.seikei.ac.jp (A.Y.)

**Keywords:** cluster analysis, constant ambient air monitoring, Kanto region, NO*_x_*, O*_x_*, non-methane hydrocarbon, suspended particulate matter

## Abstract

This study demonstrates an application of cluster analysis to constant ambient air monitoring data of four pollutants in the Kanto region: NO*_x_*, photochemical oxidant (O*_x_*), suspended particulate matter, and non-methane hydrocarbons. Constant ambient air monitoring can provide important information about the surrounding atmospheric pollution. However, at the same time, ambient air monitoring can place a significant financial burden on some autonomous communities. Thus, it has been necessary to reduce both the number of monitoring stations and the number of chemicals monitored. To achieve this, it is necessary to identify those monitoring stations and pollutants that are least significant, while minimizing the loss of data quality and mitigating the effects on the determination of any spatial and temporal trends of the pollutants. Through employing cluster analysis, it was established that the ambient monitoring stations in the Kanto region could be clustered topologically for NO*_x_* and O*_x_* into eight groups. From the results of this analysis, it was possible to identify the similarities in site characteristics and pollutant behaviors.

## 1. Introduction

Constant ambient air monitoring can provide important information about surrounding atmospheric pollution. In Japan, constant ambient air monitoring of six priority substances (SO_2_, carbon monoxide (CO), NO*_x_*, photochemical oxidant (O*_x_*), suspended particulate matter (SPM), and non-methane hydrocarbons (NMHC)) is conducted by local prefectural governments under the Air Pollution Control Act. These constant ambient air monitoring data are very useful for analyzing the current situation and trends of pollution within an area, and many research works in Japan that have used these data have been reported [1,2,3]. However, at the same time, ambient air monitoring places a significant financial burden on the autonomous communities. Thus, it is necessary to identify less significant monitoring stations and pollutants, while minimizing the loss of data quality and mitigating the effects on the determination of any spatial and temporal trends of the pollutants. There have been some trials to re-examine the efficacy of constant ambient air monitoring stations, for example, in the cities of Shizuoka, Funabashi, and Hiroshima Prefecture in Japan. However, currently, no reliable guidelines exist regarding the optimal method by which this could be achieved.

In this study, we applied cluster analysis to constant ambient air monitoring data from the Kanto region of Japan, based on the expectation that similarities in site characteristics and pollutant behaviors could be identified, and that monitoring stations could be grouped topologically.

Cluster analysis [4] and principal components analysis have been commonly used in air pollution studies. In particular, intensive cluster analyses for ozone (O_3_) and particulate matter (PM) pollution have been conducted. Lavecchia *et al.* [5] applied a cluster analysis to Italian ozone monitoring network data and discussed the similarities between the data of each monitoring station. Gramsch *et al.* [6] applied the same approach to O_3_ and PM_10_ concentrations and demonstrated that these two pollutants had similar cluster patterns, suggesting that these pollutants’ concentrations were controlled by meteorological and topographical factors. Lu *et al.* [7] applied three different cluster analyses to PM_10_ pollution in Taiwan. Giri *et al.* [8] applied a hierarchical cluster analysis to seasonal PM_10_ data in Kathmandu, Nepal, and demonstrated that monsoon rainfall had only a limited effect on decreasing PM_10_ concentrations. Cluster analysis has also been applied to other pollutants, such as nitrogen oxides (NO*_x_*) [9,10], carbon dioxide (CO_2_) [11], sulfur dioxide (SO_2_) [9], pollen [12], and Pb [13].

**Figure 1:**
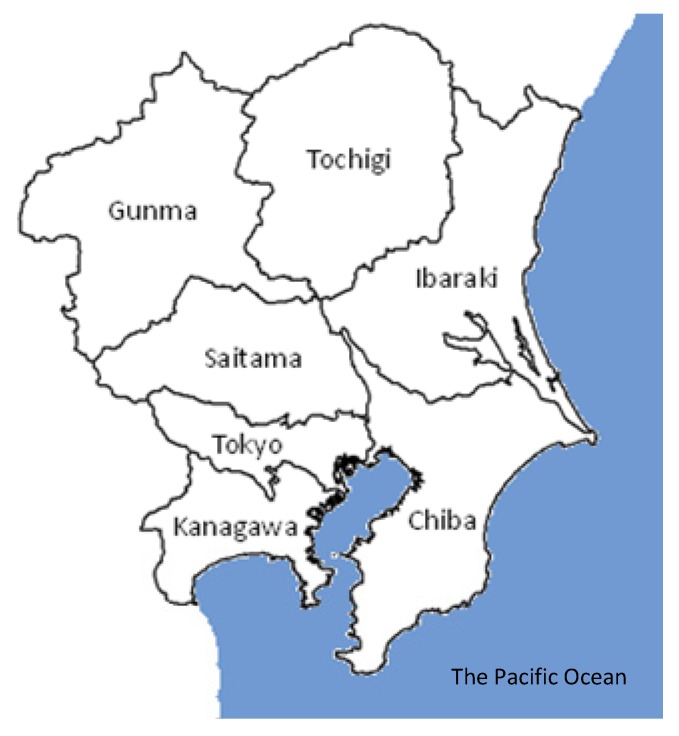
Map of the seven prefectures of the Kanto region of Japan.

We applied cluster analysis to constant ambient air monitoring data obtained in 1996 and 2006 in the Kanto region, which includes the capital region of Japan (Figure 1). By employing cluster analysis, ambient monitoring stations could be clustered topologically for NO*_x_* and O*_x_*. Based on the results of the analysis, suggestions for reducing both the number of monitoring stations and the number of chemicals monitored are possible.

## 2. Method

### 2.1. Air Monitoring Data

This study focused on the Kanto region in Japan, including the seven prefectures of Tokyo, Gunma, Tochigi, Ibaraki, Chiba, Saitama, and Kanagawa. The Kanto region has approximately 500 ambient air monitoring stations, which account for one quarter of all the monitoring stations in Japan. The constant ambient air monitoring data obtained by 476 monitoring stations during the fiscal years of 1996 and 2006 were used in this study. The monitoring data were kindly provided by the National Institute for Environmental Studies of Japan, and the data for the Kanagawa Prefecture were obtained from the prefecture’s website. The priority pollutants in this study were NO*_x_*, O*_x_*, NMHC, and SPM. SO_2_ and CO were excluded because their concentrations in the area of interest were very low and did not show large spatial differences. There are two types of ambient air monitoring stations in Japan: general environmental air monitoring stations and vehicle emission monitoring stations [14]. The latter are arranged near large roadways to monitor air pollution caused by vehicle emissions. Monitoring data from both types of station were used for the cluster analysis.

### 2.2. Cluster Analysis

SPSS^®^ Statistics 17.0 (SPSS Inc., Chicago, IL, U.S.) was used for the cluster analysis. The data matrices for each pollutant were prepared for cluster analysis using data from the air monitoring stations. In each matrix, the element in the *i*th row and jth column stands for the *i*th measurement in the year from the *j*th air monitoring station. Missing data in the *j*th column were interpolated from the annual average value of the jth monitoring station. The percentages of missing data ranged from 1.42% to 7.84%. The cluster number was fixed at eight by considering the total number of both air monitoring stations and prefecture numbers in the Kanto region. The square Euclidean distance technique and the Ward method were adopted for the cluster analysis.

### 2.3. Concentration Contour Maps

Concentration contour maps were used to clarify the characteristics of each cluster. A concentration contour map is a contour of pollutant concentrations in which the monitoring month is presented as the abscissa and the monitoring time as the ordinate. Tarasova *et al.* [15] and Zvyagintsev *et al.* [16] have used this method for air pollution analysis. Using these maps, the seasonal and diurnal variations of pollutant concentrations can be examined visually.

## 3. Results and Discussion

### 3.1. NO_x_

#### 3.1.1. General Environmental Air Monitoring Stations

**Figure 2:**
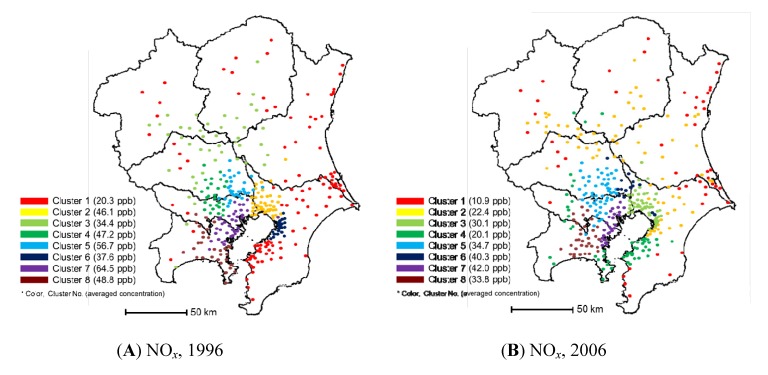
Results of clustering using NO*_x_* monitoring data from the general environmental air monitoring stations: (**A**) in 1996 and (**B**) in 2006.

For NO*_x_*, the concentration data from the general environmental air monitoring stations and vehicle emission monitoring stations were treated separately. The general environmental air monitoring stations in the Kanto region were clustered into eight groups for NO*_x_* monitoring in both fiscal years of 1996 and 2006 (Figure 2). In the legend of Figure 2, the annual average NO*_x_* concentrations for each cluster are shown. The annual average NO*_x_* concentration in the Kanto region in 1996 was 39.4 ppb, which decreased to 26.7 ppb in 2006. The annual average values were calculated by averaging all measured NO*_x_* values in the year. This concentration decrease was most likely because of the Automobile NO*_x_* PM Control Law of Japan established in 2006. By application of cluster analysis to the NO*_x_* data from the general environmental air monitoring stations, these monitoring stations were clustered territorially. Furthermore, the territorial grouping of the monitoring stations was retained. For example, cluster 7 in 1996, which shows the highest averaged NO*_x_* concentration (64.5 ppb), also appears as cluster 7 in 2006 in the same region, also with the highest NO*_x_* concentration (42.0 ppb). Clusters 4 and 5 in 1996 appear as clusters 5 and 6 in 2006 in the same region. These results show the possibility for the reasonable elimination of NO*_x_* monitoring in the general environmental air monitoring stations in such clusters, because it can be said that the air pollution by NO*_x_* in these regions is similar and that the pollution situation has not changed during the 10-year interval. Clusters with the highest average NO*_x_* concentrations, such as clusters 6 and 7 in 1996, were located in the Tokyo, Kanagawa, and Chiba prefectures, where traffic volumes were highest.

**Figure 3:**
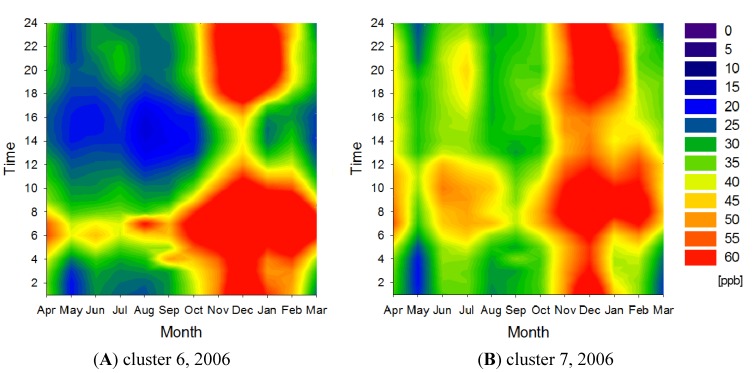
NO*_x_* concentration contour maps for clusters 6 (**A**) and 7 (**B**) in 2006.

Figure 3 presents NO*_x_* concentration contour maps for these two clusters. While the absolute concentrations are different for each cluster, all eight clusters show similar seasonal and diurnal concentration variations with two diurnal peaks at around 08:00 and 20:00, and a seasonal peak during the winter. The NO*_x_* concentration contour maps in 1996 show a similar trend to those in 2006 (data not shown). The diurnal variation in NO*_x_* concentrations can be explained partially by variations in traffic volume. However, the traffic volume on national roads in the Kanto region begins to increase during the early morning at around 04:00 and remains almost constant from 07:00 to 18:00 [17]. Therefore, variations in traffic volume cannot explain fully the decreasing NO*_x_* concentrations during the daytime. Other factors, such as NO*_x_* elimination by convection or photochemical reactions during the daytime, should also be considered in explaining the diurnal variations in NO*_x_* concentrations. The seasonal variation in NO*_x_* concentrations can be explained by the development of ground-based inversion layers during the winter, which effectively trap NO*_x_* near the ground surface. Furthermore, the strength of ultraviolet light is weaker during the winter, increasing the lifetime of NO*_x_* because of the low concentrations of hydroxyl radicals.

#### 3.1.2. Vehicle Emission Monitoring Stations

**Figure 4:**
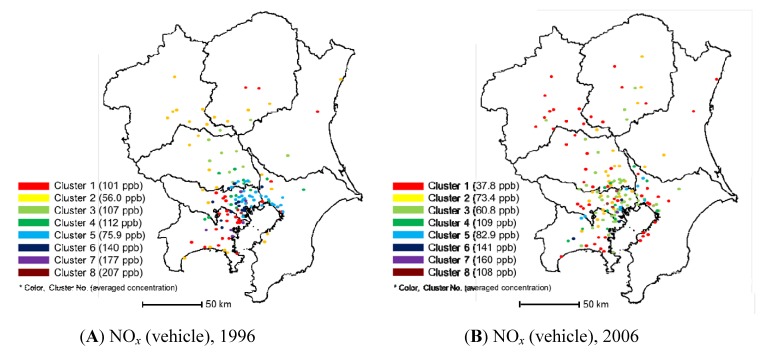
Results of clustering using NO*_x_* monitoring data from the vehicle emission monitoring stations: (**A**) in 1996 and (**B**) in 2006.

Vehicle emission monitoring stations in the Kanto region are not well classified into territorial groups for NO*_x_* monitoring in 1996 and 2006 (Figure 4), which is reasonable because the monitoring stations were installed near large roads to monitor regional air pollution from vehicular emissions. Very small clusters containing less than 10 air monitoring stations were identified. The air monitoring stations included in such small clusters show unusual NO*_x_* concentration variations. For example, the vehicle emission monitoring station at Matsubarabashi on the circular Route Kanjo 7 comprised one cluster on its own in 2006. Vehicle emissions make this road one of Japan’s most polluted areas. In this area, the Ministry of Land, Infrastructure and Transport of Japan and the Tokyo Metropolitan Government conducted air clarification experiments from 2003 to 2005. It is interesting that this highly polluted area could be extracted automatically during the cluster analysis. During the decade, the annual average NO*_x_* concentration at the vehicle emission monitoring stations also decreased from 96.3 ppb in 1996 to 63.1 ppb in 2006.

### 3.2. O_x_

**Figure 5:**
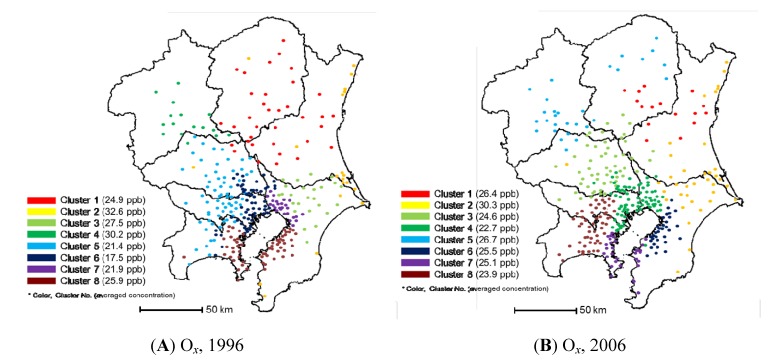
Results of clustering using O*_x_* monitoring data: (**A**) in 1996 and (**B**) in 2006.

Figure 5 presents the clustering results for air monitoring stations using O*_x_* concentrations. O*_x_* is defined as oxidative chemicals (limited to those that can generate iodine from neutralized potassium iodide solution, except for NO_2_) derived from photochemical reactions from ozone, peroxyacetyl nitrate, and others by the Ministry of the Environment of Japan. Similar to the case for NO*_x_* measured by the general environmental air monitoring stations, air monitoring stations in the Kanto region were clustered well territorially for O*_x_*_._ Despite some elimination and consolidation, the territorial group was preserved after 10 years. These results show the possibility for the reasonable elimination of O*_x_* monitoring in the air monitoring stations.

Variations in the annual average O*x* concentration in each area were small, and it is speculated that the clusters were grouped by concentration variations or other factors (e.g., time variation trends), rather than by absolute concentrations. The annual average O*_x_* concentrations in the Kanto region in 1996 and 2006 were 23.6 and 25.1 ppb, respectively.

**Figure 6:**
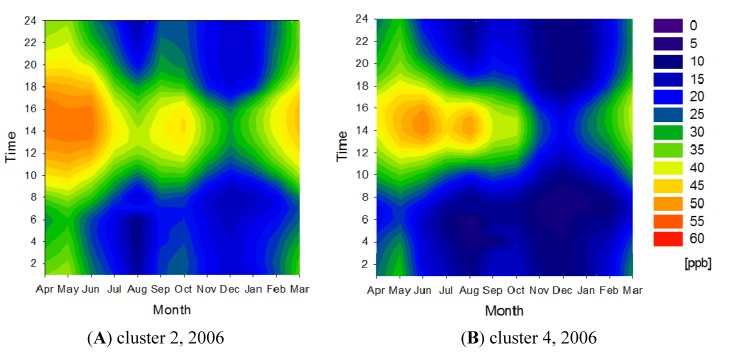
O*x* concentration contour maps for clusters 2 (**A**) and 4 (**B**) in 2006.

Figure 6 shows the concentration contour maps for clusters 2 and 4 in 2006. In 2006, cluster 2 had the highest annual average O*_x_* concentration, but much higher O*_x_* concentrations exceeding the environmental standards in Japan (60 ppb) were not observed regularly. The high annual average O*_x_* concentration in the cluster was attributed to the higher O*_x_* concentrations measured during the winter compared with other clusters. In 2006, cluster 4 had the lowest annual average O*_x_* concentration.

In cluster 3, in 2006, O*_x_* concentrations higher than the environmental standards (60 ppb) were observed more frequently, despite the cluster having an annual average concentration that could be considered normal. Cluster 3 was located in Saitama Prefecture, but also encompassed parts of the Tochigi and Ibaraki prefectures.

### 3.3. NMHC

**Figure 7:**
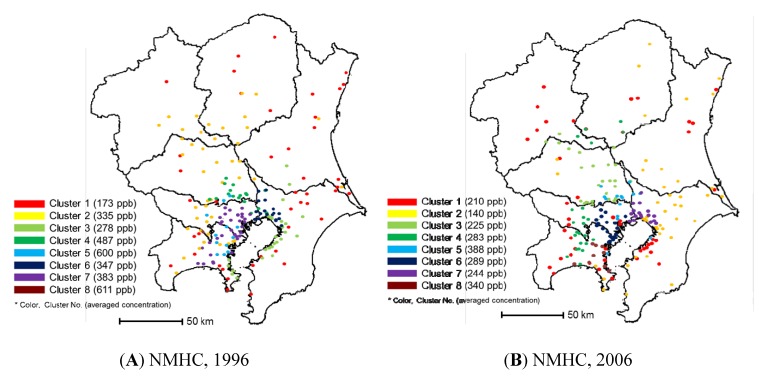
Results of clustering using NMHC monitoring data: (**A**) in 1996 and (**B**) in 2006.

Figure 7 presents the cluster analysis results using NMHC concentrations. In 1996, the Kanto region was divided approximately into two areas: an urban area and a surrounding suburban area. However, the division is not obvious because NMHC concentrations are affected by local pollution sources, similar to the clustering of NO*_x_* concentrations at the vehicle emission monitoring stations. The terrestrial division became more ambiguous in 2006. It is evident that NMHC pollution changed in the East Kanto area over the decade. These results indicate that it is difficult to eliminate monitoring stations for NMHC by the cluster method. The annual average NMHC concentration in the Kanto region decreased from 331.6 ppb in 1996 to 233.9 ppb in 2006. The percentage of vehicle emission monitoring stations was especially high in clusters 5 and 8, where the average annual NMHC concentrations were high. This suggests that vehicle emissions influence NMHC pollution within the region.

**Figure 8:**
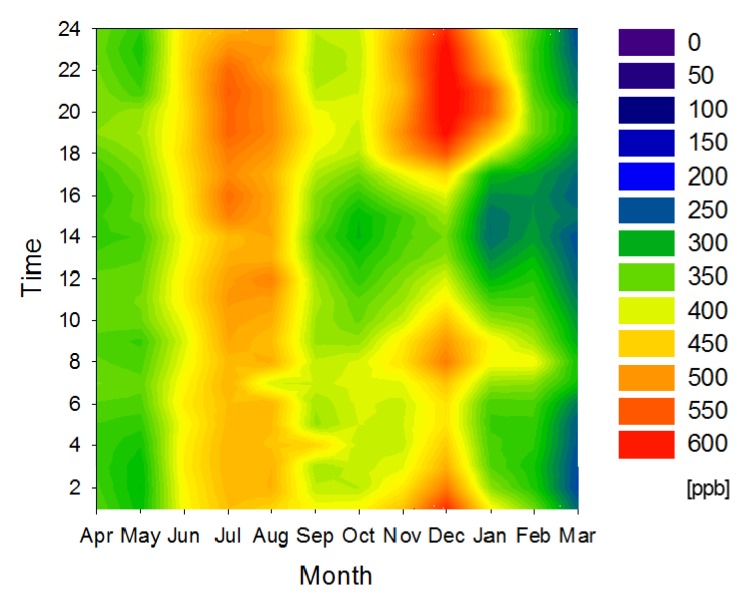
NMHC concentration contour map for cluster 5 in 2006.

Figure 8 shows the NMHC concentration contour map for cluster 5 in 2006, which experienced the highest average concentration (388 ppb). Similar to other contour maps, two peaks of NMHC concentration can be observed in many areas during the summer and winter. One possible reason for the higher NMHC concentration in the summer season could be the enhanced emissions and atmospheric chemical reactions under higher temperatures. Peak NMHC concentrations during the winter can be explained by the development of ground-level inversion layers trapping NMHC emissions.

### 3.4. SPM

**Figure 9:**
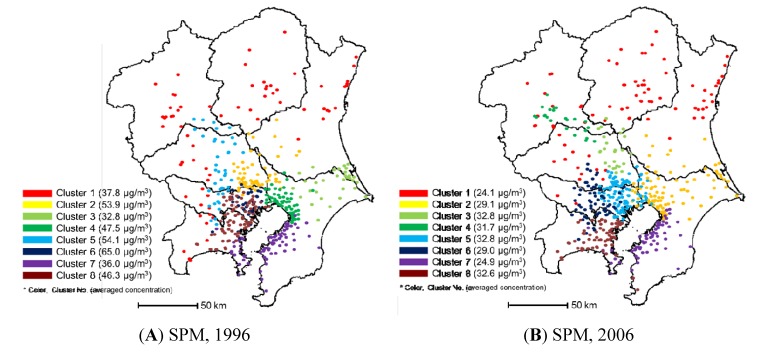
Results of clustering using SPM monitoring data: (**A**) in 1996 and (**B**) in 2006.

Figure 9 shows the cluster analysis results of air monitoring stations using SPM concentrations. In the central urban area, some overlaps of clusters were observed. Overall, the air monitoring stations in the Kanto region were clustered into eight territorial groups for both years. Some differences among the clustering for both years were observed. Clusters 2 and 3 in 1996, in the north section of Chiba, merged into one cluster (cluster 2) in 2006. Cluster 5 in 1996, split into three groups: clusters 3, 4, and 6. These results indicate that the air pollution situation changed in the Kanto region during the decade. In addition, it can be said that it is difficult to eliminate monitoring stations for SPM using the cluster method. The annual average SPM concentration in the Kanto region in 1996 was 45.5 µg/m^3^, which decreased to 29.3 µg/m^3^ in 2006. This large decrease in concentration was mainly caused by the implementation of regulation according to the Automobile NO*_x_* PM Control Law of Japan. The banning of small incinerators to prevent dioxin emissions also contributed to the decrease in SPM concentrations. The annual average SPM concentrations were highest in clusters 2, 5, and 6. The percentages of vehicle emission monitoring stations in these clusters were high. Similar to NO*_x_*, if the monitoring data from the vehicle emission monitoring stations were treated separately, the SPM pollution characteristics in the Kanto region would be clearer.

**Figure 10:**
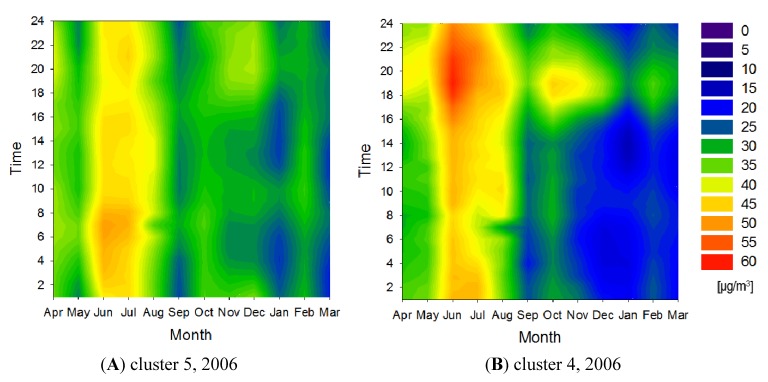
SPM concentration contour maps for clusters 5 (**A**) and 4 (**B**) in 2006.

Figure 10 presents the SPM concentration contour maps for clusters 5 and 4 in 2006. In 2006, SPM pollution during the summer was prominent in all eight clusters. The SPM concentration contour map for clusters 4 and 5 showed the typical concentration variation trend observed in the Kanto region; a concentration peak in the morning from June to July (the rainy season) and a concentration peak in the evening in December. High concentrations of SPM during the summer can be explained by accelerated photochemical reactions. Kaneyasu *et al.* [18] reported that high SPM concentrations during the rainy season in Japan could also be caused by meteorological factors. In 2006, cluster 4, located in the northern area of Saitama to the center of Gunma, showed specific SPM concentration variations, *i.e.*, they increased in the evening during the summer. In this area, southeasterly winds are dominant during the summer, suggesting that winds transported NO*_x_* and NMHC that were emitted in the urban area during the day. Photochemical reactions occurring during their transport would contribute to the elevated SPM concentrations in cluster 4 in the evening.

## 4. Conclusions

Ambient air monitoring stations in the Kanto region were clustered into eight groups using constant air monitoring concentrations of NO*_x_*, O*_x_*, NMHC, and SPM. The air pollution characteristics of the clusters were analyzed using concentration contour maps. It was confirmed that the ambient monitoring stations could be clustered topologically for NO*_x_* and O*_x_* using cluster analysis. If the ambient air monitoring stations could be reasonably grouped, then a method for reducing the number of both monitoring stations and chemicals monitored should be possible. Such a method should be simple, versatile, and mechanical and thus, we suggest that the number of monitoring stations in the Kanto region could be reduced by adopting the following three simple criteria: (1) retain the monitoring station (or chemical) if similarities exist between its monitored data and the averaged monitored data of the cluster to which it belongs; (2) retain the monitoring station (or chemical) if the monitored data show higher concentrations; and (3) retain the monitoring station (or chemical) if the monitored concentration levels exhibit an increasing trend. For the first criterion, Euclidean distances between each element of monitored data and the average of the monitored data in the topological group matrix were calculated, and only the top 5%–15% of monitoring stations with the smallest Euclidean distances was retained. For the second criterion, the top 5%–15% of monitoring stations with the highest annual averaged concentrations in 1996 or 2006 was retained. For the third criterion, the top 5%–15% of monitoring stations with the highest ratio of annual averaged concentrations in 2006 to 1996 was retained. The retention ratio for each criterion was varied within the range of 5%–15%. When the retention ratio was set at 10%, over 30% of monitoring stations could be removed by adopting the above criteria. In our next paper, we will describe this suggested method in greater detail.

## References

[1] Ito A., Hirai H., Morikawa T., Hayashi S. (2007). Application of multiple regression analysis to pollutant concentrations from roadside and ambient air quality monitoring stations. JARI Res. J..

[2] Shimizu G., Yoshizawa H., Ishii H., Takahashi T., Hidaka T., Yamamoto T., Kotorii S., Horiuchi T. (2006). Alteration of the SPM pollution in Pref.Yamanashi compared with the south Kanto area. Annu. Rep. Yamanashi Inst. Public Health.

[3] Shimizu G., Egashira K., Chisuwa S. (2008). Results of air pollution monitoring for 1988–2007 in Pref. Yamanashi. Annu. Rep. Yamanashi Inst. Public Health.

[4] Everitt B.S. (1993). Cluster Analysis.

[5] Lavecchia C., Angelino E., Bedogni M., Bravetti E., Gualdi R., Lanzani G., Musitelli A., Valentini M. (1996). The ozone patterns in the aerological basin of Milan (Italy). Environ. Software.

[6] Gramsch E., Cereceda-Balic F., Oyola P., Baer D. (2006). Examination of pollution trends in Santiago de Chile with cluster analysis of PM_10_ and ozone data. Atmos. Environ..

[7] Lu H.-C., Chang C.-L., Hsieh J.-C. (2006). Classification of PM_10_ distributions in Taiwan. Atmos. Environ..

[8] Giri D., Murthy V.K., Adhikary P.R., Khanal S.N. (2007). Cluster analysis applied to atmospheric PM_10_ concentration data for determination of sources and spatial patterns in ambient air-quality of Kathmandu valley. Curr. Sci..

[9] Saksena S., Joshi V., Patil R.S. (2003). Cluster analysis of Delhi's ambient air quality data. J. Environ. Monit..

[10] Ignaccolo R., Ghigo S., Giovenali E. (2008). Analysis of air quality monitoring networks by functional clustering. Environmetrics.

[11] Eneroth K., Aalto T., Hatakka J., Holmen K., Laurila T., Viisanen Y. (2005). Atmospheric transport of carbon dioxide to a baseline monitoring station in northern Finland. Tellus.

[12] Rieux C., Personnaz M.-B., Thibaudon M. (2008). Spatial variation of airborne pollen over south-east France: Characterization and implications for monitoring networks management. Aerobiologia.

[13] Sârbu C., Einax J.W. (2008). Study of traffic-emitted lead pollution of soil and plants using different fuzzy clustering algorithms. Anal. Bioanal. Chem..

[14] Ministry of the Environment of Japan (2007). Manual for Environmental Air Constant Monitoring.

[15] Tarasova O.A., Brenninkmeijer C.A.M., Jockel P., Zvyagintsev A.M., Kuznetsov G.I. (2007). A climatology of surface ozone in the extra tropics: Cluster analysis of observations and model results. Atmos. Chem. Phys..

[16] Zvyagintsev A.M., Tarasova O.A., Kuznetsov G.I. (2007). Seasonal and daily cycles of surface ozone in the extratropical latitudes. Izv. Atmos. Ocean. Phys..

[17] The Ministry of Land, Infrastructure and Transport, Kanto Regional Development Bureau Road Investor Relations Site. http://www.mlit.go.jp/road/ir/ir-data/ir-data.html.

[18] Kaneyasu N., Suzuki M., Sugimoto N., Matsui I., Shimizu A. (2005). Meteorological factors which causes high SPM concentrations in wide area in rainy season—Analysis of the case in July 1997. Earozoru Kenkyu.

